# HIV-1 Evolutionary Dynamics under Nonsuppressive Antiretroviral Therapy

**DOI:** 10.1128/mbio.00269-22

**Published:** 2022-04-21

**Authors:** Steven A. Kemp, Oscar J. Charles, Anne Derache, Werner Smidt, Darren P. Martin, Collins Iwuji, John Adamson, Katya Govender, Tulio de Oliveira, Francois Dabis, Deenan Pillay, Richard A. Goldstein, Ravindra K. Gupta

**Affiliations:** a Cambridge Institute of Therapeutic Immunology & Infectious Disease (CITIID), University of Cambridge, Cambridge, United Kingdom; b Division of Infection & Immunity, University College London, London, United Kingdom; c Africa Health Research Institute, Durban, South Africa; d Department of Integrative Biomedical Sciences, University of Cape Town, Cape Town, South Africa; e Research Department of Infection and Population Health, University College London, United Kingdom; f KRISP - KwaZulu-Natal Research and Innovation Sequencing Platform, UKZN, Durban, South Africa; g INSERM U1219-Centre Inserm Bordeaux Population Health, Université de Bordeaux, France; h Université de Bordeaux, ISPED, Centre INSERM U1219-Bordeaux Population Health, France; U.S. Military HIV Research Program; HJF; University of Pittsburgh School of Medicine

**Keywords:** antiretroviral resistance, clinical failure, drug resistance evolution, human immunodeficiency virus

## Abstract

Prolonged virologic failure on 2nd-line protease inhibitor (PI)-based antiretroviral therapy (ART) without emergence of major protease mutations is well recognized and provides an opportunity to study within-host evolution in long-term viremic individuals. Using next-generation sequencing and *in silico* haplotype reconstruction, we analyzed whole-genome sequences from longitudinal plasma samples of eight chronically infected HIV-1-positive individuals failing 2nd-line regimens from the French National Agency for AIDS and Viral Hepatitis Research (ANRS) 12249 Treatment as Prevention (TasP) trial. On nonsuppressive ART, there were large fluctuations in synonymous and nonsynonymous variant frequencies despite stable viremia. Reconstructed haplotypes provided evidence for selective sweeps during periods of partial adherence, and viral haplotype competition, during periods of low drug exposure. Drug resistance mutations in reverse transcriptase (RT) were used as markers of viral haplotypes in the reservoir, and their distribution over time indicated recombination. We independently observed linkage disequilibrium decay, indicative of recombination. These data highlight dramatic changes in virus population structure that occur during stable viremia under nonsuppressive ART.

## INTRODUCTION

Even though HIV-1 infections are most commonly initiated with a single founder virus (1), acute and chronic disease are characterized by extensive inter- and intrapatient genetic diversity (2, 3). The rate and degree of diversification is influenced by multiple factors, including selection pressures imposed by the adaptive immune system, exposure of the virus to drugs, and tropism/fitness constraints relating to replication and cell-to-cell transmission in different tissue compartments (4, 5). During HIV-1 infection, high rates of reverse transcriptase (RT)-related mutation and high viral turnover during replication result in swarms of genetically diverse variants (6) which coexist as quasispecies (7, 8). The existing literature on HIV-1 intrahost population dynamics is largely limited to untreated infection, in subtype B-infected individuals (9–12). These works have shown nonlinear diversification of virus both toward and away from the founder strain during chronic untreated infection.

Viral population dynamics in long-term viremic antiretroviral therapy (ART)-treated individuals have not been characterized. HIV-1 rapidly accumulates drug resistance-associated mutations (DRMs), particularly during nonsuppressive 1st-line ART (5, 13). As a result, ART-experienced patients failing 1st-line regimens for prolonged periods of time are characterized by high frequencies of common nucleoside reverse transcriptase (NRTI) and nonnucleoside reverse transcriptase (NNRTI) DRMs such as M184V, K65R, and K103N (14). Routinely, 2nd-line ART regimens consist of two NRTIs in conjunction with a boosted protease inhibitor (PI). Although PI DRMs are uncommonly reported (15), a situation that differs for less potent drugs used in the early PI era (5), multiple studies have indicated that diverse mutations accumulating in the *gag* gene during PI failure might impact PI susceptibility (16–22). Common pathways for these diverse mutations have, however, been difficult to discern, likely reflecting multiple routes to drug escape.

Prolonged virological failure on PI-based regimens without the emergence of PI DRMs provides an opportunity to study evolution under partially suppressive ART. The process of selective sweeps in the context of HIV-1 infection has previously been described (23, 24). Although major PI DRMs and other nonsynonymous mutations in regulatory regions such as *pol* can significantly lower fitness (2, 25, 26), these studies typically are oblivious to temporal sequencing.

We have deployed next-generation sequencing of stored blood plasma specimens from patients in the Treatment as Prevention (TasP) French National Agency for AIDS and Viral Hepatitis Research (ANRS) 12249 study (27), conducted in Kwazulu-Natal, South Africa. All patients were infected with HIV-1 subtype C and characterized as failing 2nd-line regimens containing lopinavir and ritonavir (LPV/r), with prolonged virological failure in the absence of major known PI mutations (28). In the manuscript, we report the details of evolutionary dynamics during nonsuppressive 2nd-line ART. By sampling patients consistently over 2 or more years, we propose that ongoing evolution is driven by the dynamic flux between genetic drift, fitness-driven selection, and recombination, exemplified by resistance mutations that have undergone reassortment across haplotypes through recombination.

## RESULTS

### Patient characteristics.

Eight south African patients with virological failure of 2nd-line PI-based ART, with between three and eight time points and viremia of >1,000 copies/mL, were selected from the French ANRS TasP trial for viral dynamic analysis. Collected patient metadata included viral loads, regimens, and time since ART initiation (Table 1). HIV RNA was isolated from venous blood samples and subjected to whole-genome sequencing (WGS) using Illumina technology; from this, whole-genome haplotypes were reconstructed using sites with a depth of ≥100 reads (Fig. S1). Prior to participation in the TasP trial, patients accessed 1st-line regimens for an average of 5.6 years (±2.7 years). At baseline enrollment into TasP (while failing 1st-line regimens), the median patient viral load was 4.96 × 10^10^ copies/mL (interquartile range [IQR], 4.17 × 10^10^ to 5.15 × 10^10^); 12 DRMs were found at a threshold of >2%, the most common of which were the RT mutations, K103N, M184V, and P225H, which are consistent with previous use of stavudine (d4T), nevirapine (NVP), efavirenz (EFV), and emtricitabine/lamivudine (FTC/3TC). Six of the eight patients had minority frequency DRMs associated with PI failure (average, 6.4%) which were usually seen only in one sample per patient throughout the longitudinal sampling. The observed mutations included L23I, I47V, M46I/L, G73S, V82A, N83D, and I85V (Tables S1a to 3c). The viral populations of four of the eight patients also carried major integrase strand inhibitor (INSTI) mutations, also at minority frequencies (average, 5.0%) and also usually at single time points (T97A, E138K, Y143H, Q148K). Of note, patients were maintained on protease inhibitors during viremia, as poor adherence was suspected as the reason for ongoing failure. Sanger sequencing of all subtype C viruses was undertaken during routine clinical monitoring and was consistent with next-generation sequencing (NGS) data (Tables S1a to 3c) regarding the absence of PI DRMS.

**TABLE 1:** Regimens and viral load at the final time point for all patients^*a*^^,^^*b*^

Patient	No. of time points	1st-line regimen	Time since initiation of 1^st^-line treatment (yrs)	2^nd^-line regimen	Viral load at final time point (copies/mL)
15664	6	d4T, 3TC, FTC	6.2	LPV/r, TDF, FTC	28,655
16207	5	d4T, 3TC, NVP	5.9	LPV/r, TDF, FTC	56,660
22763	8	d4T, 3TC, EFV	6.2	LPV/r, TDF, 3TC	15,017
22828	6	d4T, 3TC, NVP	6.4	LPV/r, TDF, 3TC/FTC	947
26892	7	d4T, 3TC, EFV	6	LPV/r, TDF, FTC	12,221
28545	5	TDF, FTC, EFV	1.3	LPV/r, AZT, 3TC	12,964
29447	4	TDF, FTC, EFV	2.8	LPV/r, TDF, FTC	64,362
47939	3	d4T, 3TC, EFV	10.1	LPV/r, AZT, 3TC/FTC	6,328

a Patients initiated and maintained 1st-line regimens for between 1 and 10 years before being switched to 2nd-line regimens as part of the TasP trial. Eight of the nine patients were failing 2nd-line regimens at the final time point.

b NRTI: d4T, stavudine; 3TC, lamivudine; TDF, tenofovir; FTC, emtricitabine; AZT, zidovudine. NNRTI: EFV, efavirenz; NVP, nevirapine. PI: LPV/r, lopinavir/ritonavir.

10.1128/mbio.00269-22.1 FIG S1 Read depth per site for all BAM files. Any site that had coverage of <100 reads (indicated as a horizontal black line) was excluded from haplotype reconstruction and reversion to consensus calculations. Download FIG S1, PDF file, 2.1 MB. © Crown copyright 2022. 2022 Crown https://creativecommons.org/licenses/by/4.0/ This content is distributed under the terms of the Creative Commons Attribution 4.0 International license.

10.1128/mbio.00269-22.7 TABLE S1 (a) Patient 15664 *gag* variant frequencies across successive time points. For all supplemental tables, figures are in percentage of variant in a variant call format VCF at each time point. (b) Patient 15664 *pol* variants. (c) Patient 15664 *env* variants. Download Table S1, DOCX file, 0.06 MB. © Crown copyright 2022. 2022 Crown https://creativecommons.org/licenses/by/4.0/ This content is distributed under the terms of the Creative Commons Attribution 4.0 International license.

### SNP frequencies and measures of diversity/divergence over time.

**FIG 1:**
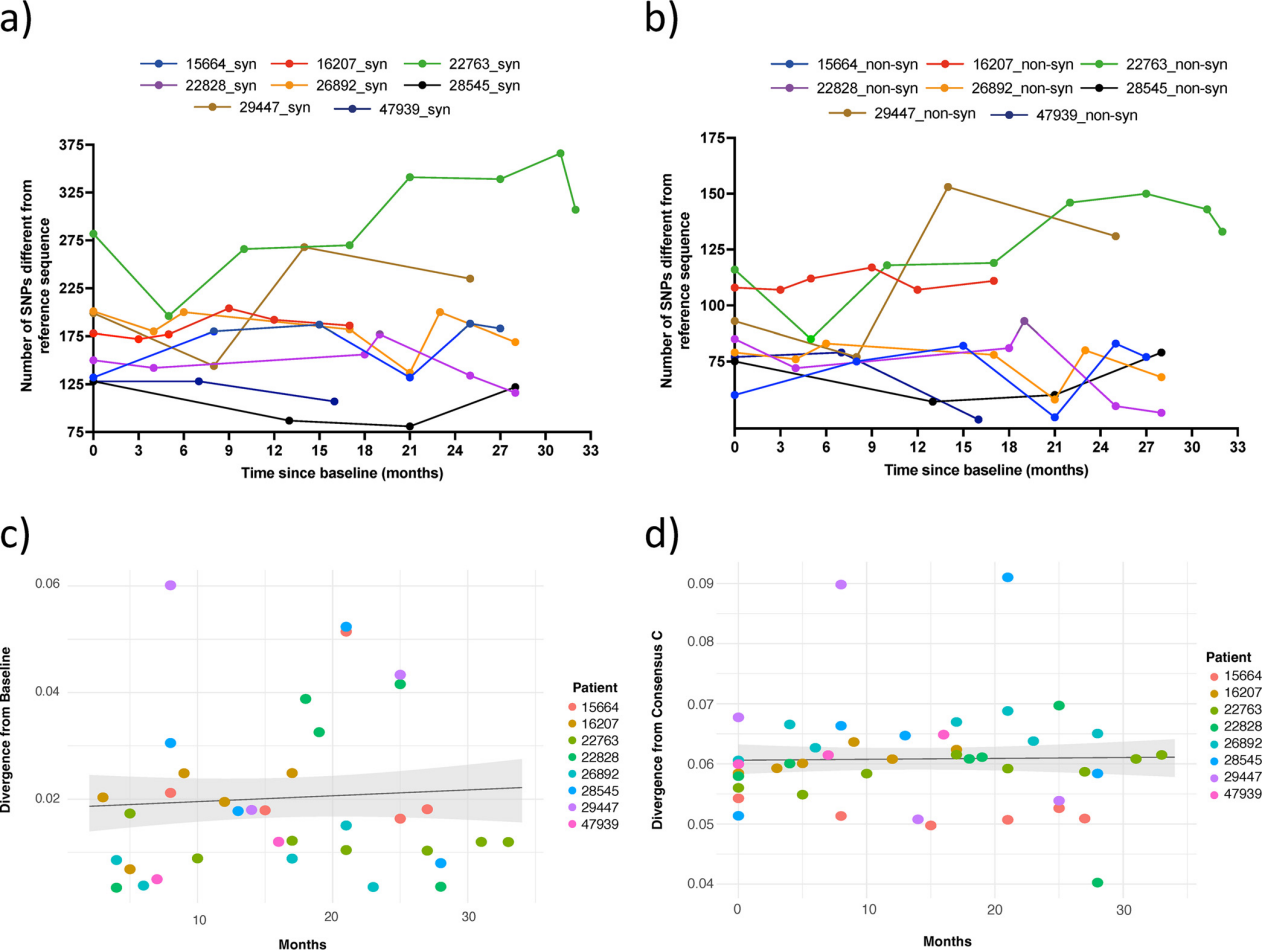
Sequence divergence for eight patients under nonsuppressive ART. These data were for SNPs detected by Illumina NGS at <2% abundance. Sites had coverage of at least 10 reads. (a and b) In both (a) synonymous and (b) nonsynonymous mutations, there was an idiosyncratic change in the number of SNPs relative to the reference strain over time. (c) Mixed-effects linear model of divergence from the baseline time point and (d) consensus C subtype. The trend and 95% confidence interval (CI) (gray shadows) suggest that there is no strong positive or negative linear relationship between divergence and time.

WGS data were used to measure the changing frequencies of viral single nucleotide polymorphisms (SNPs) relative to a dual-tropic subtype C reference sequence (GenBank accession number AF411967) within individuals over time (Fig. 1a and b). The number of longitudinal synonymous SNPs mirrored the number of nonsynonymous SNPs, but the former were 2- to 3-fold more common. Diversification was considered by counting the number of SNPs relative to the reference sequence. There were dynamic changes in the numbers of SNPs over time, with both increases and decreases in the numbers of SNPs, suggesting population competition, and/or the occurrence of selective sweeps. From time point 2 onward (all patients now on 2nd-line, PI-containing regimens for >6 months), all patients (except 28545) had increases in both synonymous and nonsynonymous SNPs.

In previous literature, viral populations within untreated, chronically infected HIV-1 patients have been shown to revert toward the founder or infecting virus states (9). We repeated this analysis with our chronically infected, but treated, HIV-1 population, considering separately the earliest consensus sequence, HIV-1 subtype C consensus, and M group consensus sequences as founder strains. Divergence from the founder strain per patient time point was measured by calculating the genetic distance between patient and founder for each longitudinal sample.

To assess if (i) there was a general trend of reversion to founder and (ii) time was an explanatory variable to that trend, we utilized a linear mixed-effects model (LMEM). Divergence from the founder was modeled as the response, each patient was treated as a random effect, and the time from first patient sample was treated as a fixed effect, “time.” Modeling the whole-genome sequences indicated that there was no significant effect of time (in months) on viral diversification or reversion to the infecting/baseline strain or on ancestral C state (Fig. 1c and d, Table S4).

10.1128/mbio.00269-22.8 TABLE S2 (a) Patient 16207 *gag* variant frequencies. (b) Patient 16207 *pol* variant frequencies. (c) Patient 16207 *env* variant frequencies. Download Table S2, DOCX file, 0.06 MB. © Crown copyright 2022. 2022 Crown https://creativecommons.org/licenses/by/4.0/ This content is distributed under the terms of the Creative Commons Attribution 4.0 International license.

10.1128/mbio.00269-22.9 TABLE S3 (a) Patient 22763 *gag* variant frequencies. (b) Patient 22763 *pol* mutations. (c) Patient 22763 *env* mutations. Download Table S3, DOCX file, 0.08 MB. © Crown copyright 2022. 2022 Crown https://creativecommons.org/licenses/by/4.0/ This content is distributed under the terms of the Creative Commons Attribution 4.0 International license.

10.1128/mbio.00269-22.10 TABLE S4 Results from linear mixed effects models of effect of months on divergence from founder virus. Download Table S4, DOCX file, 0.01 MB. © Crown copyright 2022. 2022 Crown https://creativecommons.org/licenses/by/4.0/ This content is distributed under the terms of the Creative Commons Attribution 4.0 International license.

When assessing the constituent 1,000-bp genomic regions of each alignment, four genomic regions were significant for divergence from the ancestral C state, indicating that time in months impacted viral divergence. This revealed that in portions of the genome (*pol*, *vpu*, and *env*) there was sufficient statistical support to confirm that there was ongoing divergence from the subtype C consensus. However, correction for false-discovery rate (FDR) with a Benjamini-Hochberg correction revealed that this divergence was not significant. Furthermore, analysis of amino acids on a site-by-site basis showed that AA mutations almost always resulted in a divergence, rather than a reversion toward baseline/ancestral sequences. Divergence from these ancestral sequences is likely enabled by recombination, which unlinks hyper-variable loci from strongly constrained neighboring sites. Collectively, we found no statistically significant evidence for reversions and were therefore unable to conclude that these patients are reverting to founder as described in previous literature (9, 29).

**FIG 2:**
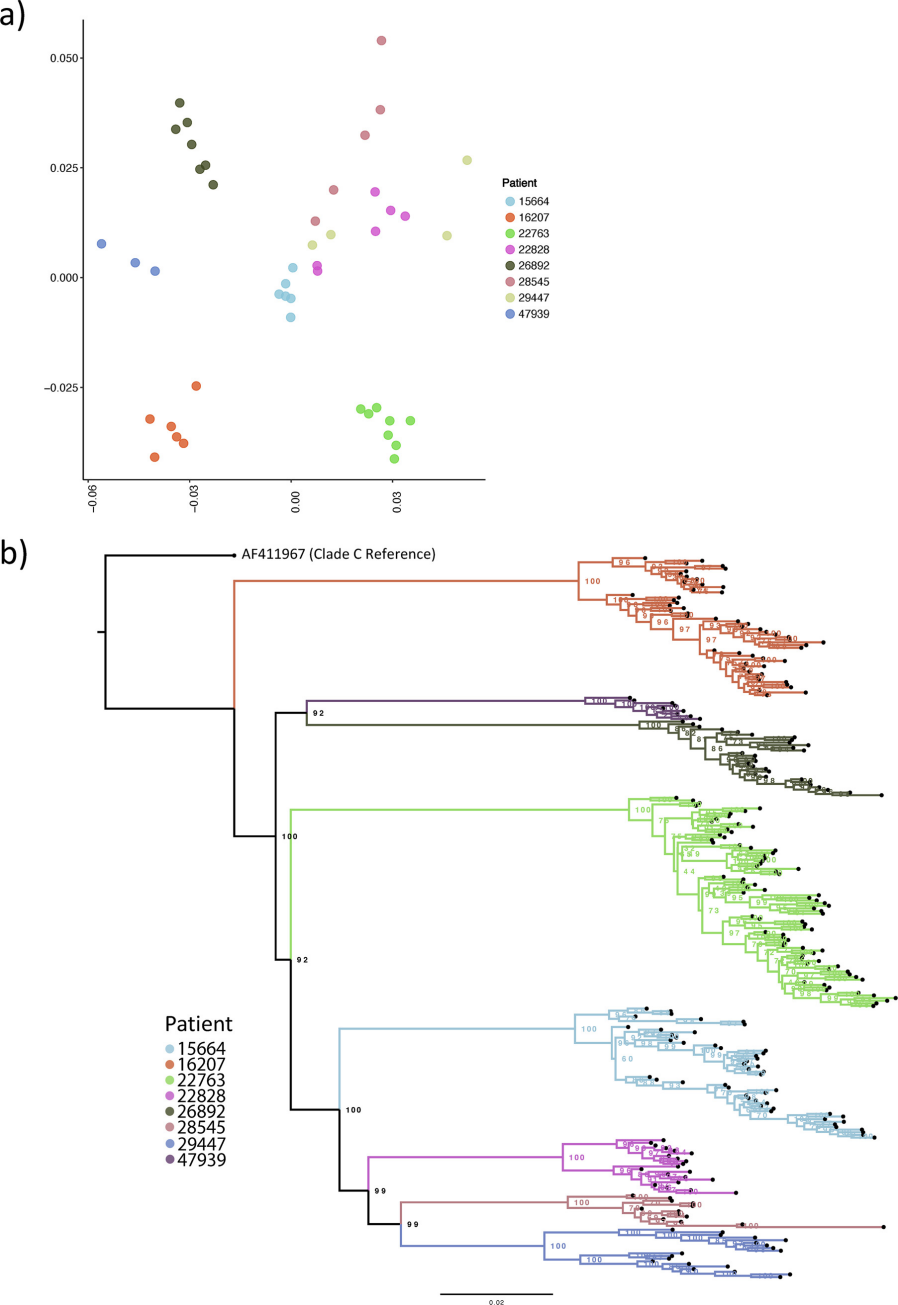
(a) Multidimensional scaling showing clustering of HIV whole genomes from consensus sequences with high intrapatient diversity. Multidimensional scaling (MDS) was created by determining all pairwise distance comparisons under a TN93 substitution model, color-coded by patient. Axes are MDS-1 and MDS-2. (b) Maximum likelihood phylogeny of constructed viral haplotypes for all patients. The phylogeny was rooted on the clade C reference genome (GenBank accession number AF411967). Reconstructed haplotypes were genetically diverse and did not typically cluster by time point.

**FIG 3:**
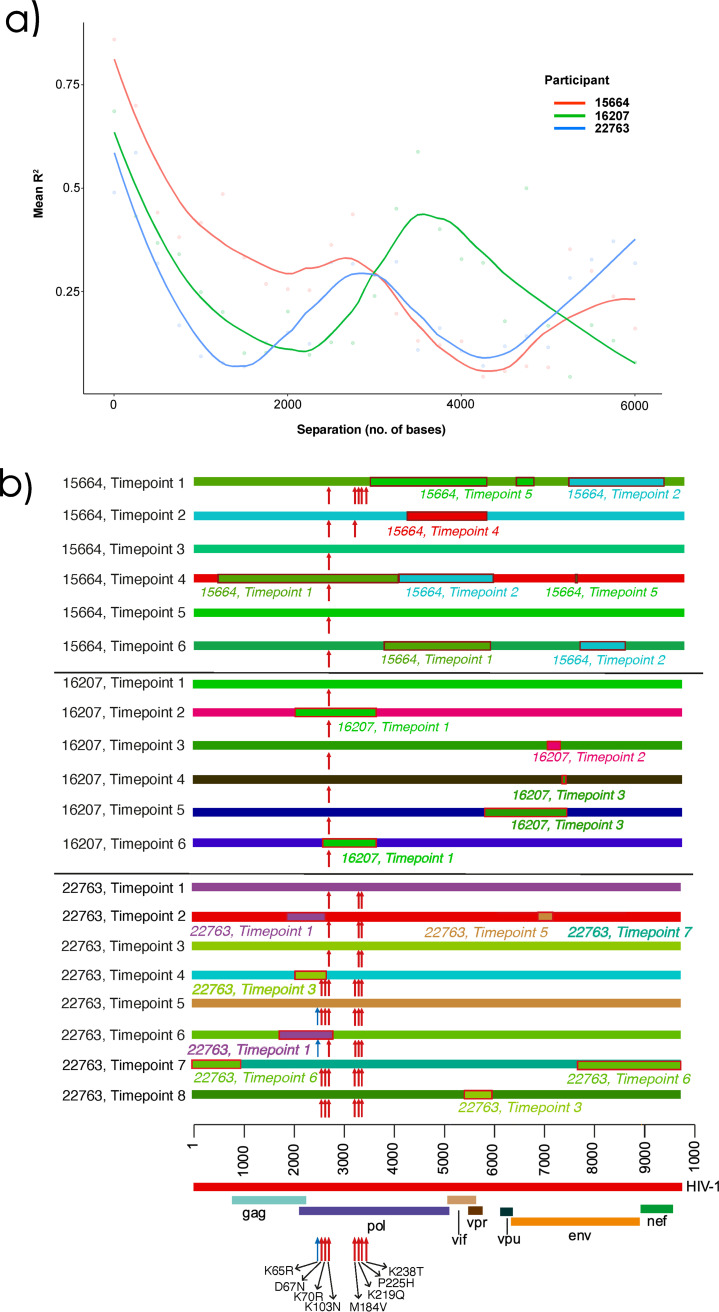
(a) Pairwise linkage disequilibrium decays rapidly with increasing distance between SNPs. Lines represent patterns of LD for each patient examined in-depth. There was a constant decrease in linkage disequilibrium over the first 800 bp. (b) Putative recombination breakpoints and drug resistance-associated mutations of all longitudinal consensus sequences belonging to three patients, 15664, 16207, and 22763. All sequences were colored uniquely; perceived recombination events supported by 4 or more methods implemented in RDP5 are highlighted with a red border and italic text to show the major parent and recombinant portion of the sequence. Drug resistance-associated mutations are indicated with a red arrow, relative to the key at the bottom of the image. For ease of distinguishment, the K65R mutations are indicated with a blue arrow.

To assess the relationship of the observed divergence patterns, we examined nucleotide diversity by considering all pairwise nucleotide distances of each consensus sequence, by time point and patient utilizing multidimensional scaling (30). Intrapatient nucleotide diversity varied considerably between patients (Fig. 2a). Viruses from some patients showed little diversity between time points (e.g., patient 16207), whereas those from others showed higher diversity between time points (e.g., patient 22763). In some instances, a patient’s viruses were tightly clustered, suggesting little change over time (Fig. 3a, patients 16207, 26892, and 47939) compared to others (patients 22828 and 28545). To corroborate the multidimensional scaling (MDS) approach, we used an alternative novel method of examining nucleotide diversity of longitudinal time points using all positional information from BAM files (Fig. S2).

10.1128/mbio.00269-22.2 FIG S2 Whole-genome nucleotide diversity of longitudinal time points from each patient. Diversity was calculated using all information from BAM files by fitting observed variant frequencies to two distributions (a β-distribution and 4D Dirichlet plus Δ function). Each dot in the scatter represents a different time point and highlights differences in whole-genome diversity between successive time points. Download FIG S2, PDF file, 0.07 MB. © Crown copyright 2022. 2022 Crown https://creativecommons.org/licenses/by/4.0/ This content is distributed under the terms of the Creative Commons Attribution 4.0 International license.

### Phylogenetic analysis of inferred haplotypes.

The preceding diversity assessments suggested the existence of distinct viral haplotypes within each patient. We therefore used a recently reported computational tool, Haplotype Reconstruction for Longitudinal Samples (HaROLD) (31), to infer 289 unique haplotypes across all patients, with between 11 and 32 haplotypes (average, 21) per patient. The number of haplotypes changed dynamically between successive time points, indicative of dynamically shifting populations (Fig. 2b). To confirm the plausibility of haplotypes, a phylogeny of all consensus sequences was inferred (Fig. S3), and an MDS plot of all viral haplotypes was constructed (Fig. S4).

10.1128/mbio.00269-22.3 FIG S3 Maximum likelihood phylogeny of consensus sequences from all time points from all patients. Phylogenies were rooted on a South African-origin subtype C reference genome (GenBank accession number AF411967). Trees were inferred with a GTR model with 1,000 rapid bootstrap replicates. Bootstrap values are indicated at all nodes. The phylogeny is consistent with the haplotype tree shown in **Fig. 2b**, indicating that haplotypes were accurate representations of sequences. Download FIG S3, PDF file, 0.1 MB. © Crown copyright 2022. 2022 Crown https://creativecommons.org/licenses/by/4.0/ This content is distributed under the terms of the Creative Commons Attribution 4.0 International license.

10.1128/mbio.00269-22.4 FIG S4 MDS scatterplot of reconstructed haplotypes. Plots were produced by obtaining a multiple sequence alignment, calculating average pairwise distances between all pairs, and then conducting multidimensional scaling under a TN93 substitution matrix. Each axis represents the component scores of the most variable axis and the second-most variable axis. Haplotypes show an increased measure of diversity compared to consensus-level variants. This is due to increased resolution of potential viral quasispecies. Download FIG S4, PDF file, 0.3 MB. © Crown copyright 2022. 2022 Crown https://creativecommons.org/licenses/by/4.0/ This content is distributed under the terms of the Creative Commons Attribution 4.0 International license.

### Linkage disequilibrium (LD) and recombination.

LD between two pairwise loci is reduced by recombination, such that LD tends to be higher for loci that are close and lower for more distant loci (32). HIV-1 is known to recombine such that sequences are not generally in linkage disequilibrium beyond 400 bp (9). The significance of recombination in an intrahost, chronic-infection setting is less well understood (33). To assess whether intrapatient recombination was occurring between the haplotypes observed in each of the three most sampled patients, we determined LD decay patterns. We assumed that if there was random recombination, this would equate to smooth LD decay patterns. This was not observed. Rather, each patient demonstrated a complex decay pattern, consistent with nonrandom recombination along the genome (Fig. 3a). Given this, we characterized recombination patterns (Fig. 3b). Inferred recombination breakpoints were identified within patients over successive time points (Fig. S5). DRMs accumulated over successive time points for patient 22763, whereas in patient 15664 the reverse was true. Patient 16207 had recombinant breakpoints localized in the *pol* gene in two time points, though it retained its majority DRM (K103N) across all haplotype populations, possibly as a result of K103N being acquired as a transmitted DRM or as all variants were under the same selective pressure.

10.1128/mbio.00269-22.5 FIG S5 Patterns of SNPs at perceived recombination breakpoint locations. In all patients where there was recombination detected, there were distinct patterns or haplotypes observable across multiple sites. Distinct patterns were observable between recombination breakpoints, lending support to the theory that recombination between different genomes was occurring, to give rise to numerous haplotypes. In numerical terms where each number represents an individual pattern (i.e., 0, 1, or 2), patient 15664 haplotype pattern 1, (A, left), assumes a 011011 distribution, pattern 2 (A, middle) as 0100010 and pattern 3 (A, right) as 000001. Patient 16207, pattern one (B, left) is 010223 and pattern 2 (B, right) is 010101. Patient 22763, pattern one (C, left) is 00011211 and pattern 2 (C, right) is 00011210. Download FIG S5, PDF file, 0.1 MB. © Crown copyright 2022. 2022 Crown https://creativecommons.org/licenses/by/4.0/ This content is distributed under the terms of the Creative Commons Attribution 4.0 International license.

### Changing landscapes of nonsynonymous and synonymous mutations.

**FIG 4:**
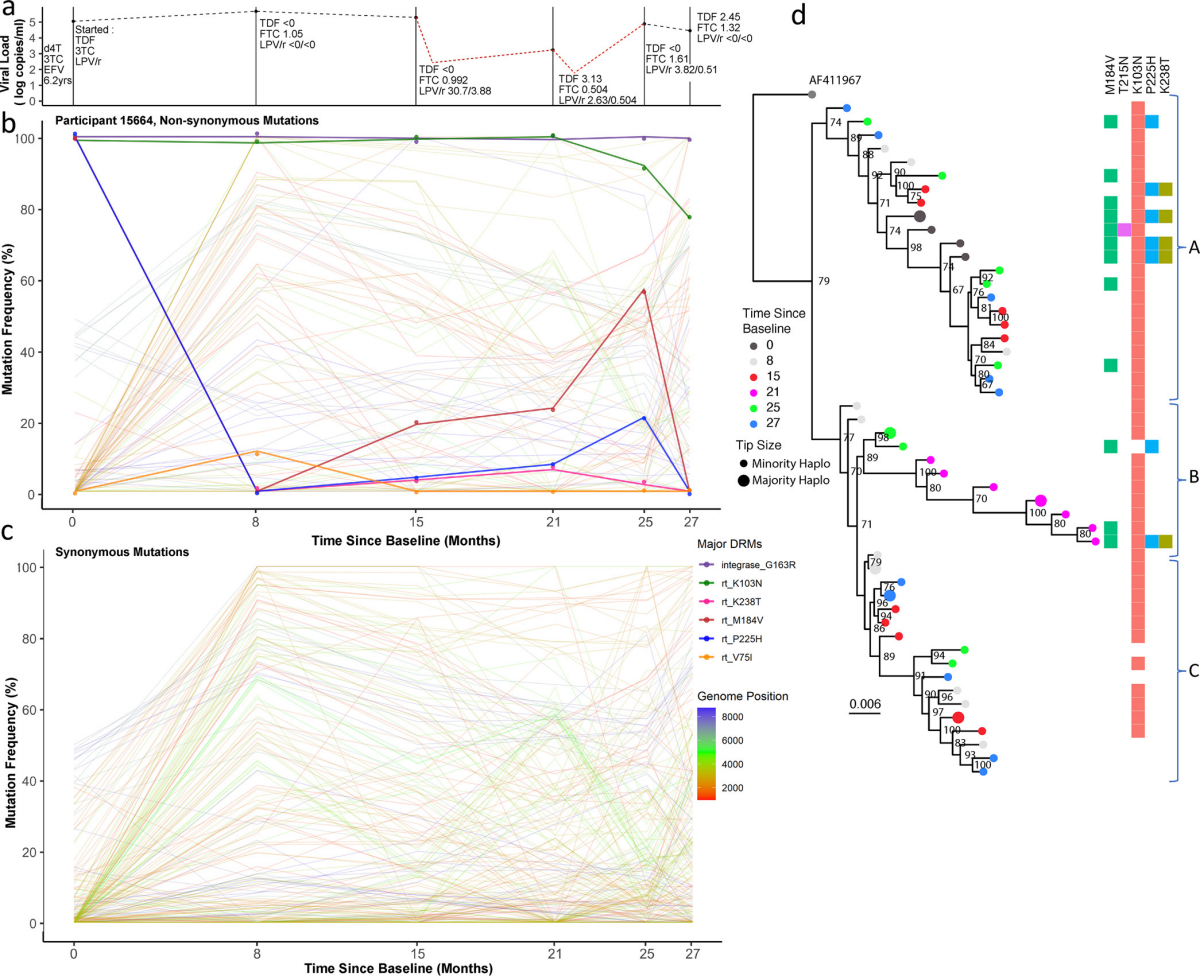
Drug regimen, adherence, and viral dynamics within patient 15664. (a) Viral load and drug levels. At successive time points the drug regimen was noted and the plasma drug concentration measured by HPLC (nmol/L). The patient was characterized by multiple partial suppression (<750 copies/mL, 16 months; <250 copies/mL, 22 months) and rebound events (red dotted line) and poor adherence to the drug regimen. (b) Drug resistance- and non-drug resistance-associated nonsynonymous mutation frequencies determined by Illumina NGS. The Patient had large population shifts between time points 1 and 2, consistent with a hard selective sweep, coincident with the shift from the 1st-line regimen to 2nd-line. (c) Synonymous mutation frequencies. All mutations with a frequency of <10% or >90% at two or more time points were tracked over successive time points. Most changes were restricted to *gag* and *pol* regions and had limited shifts in frequency, i.e., between 20 and 60%. (d) Maximum likelihood phylogeny of reconstructed haplotypes. Haplotypes largely segregated into three major clades (labeled A to C). Majority and minority haplotypes, some carrying lamivudine resistance mutation M184V. Clades referred to in the text body are shown to the right of the heatmap.

**FIG 5:**
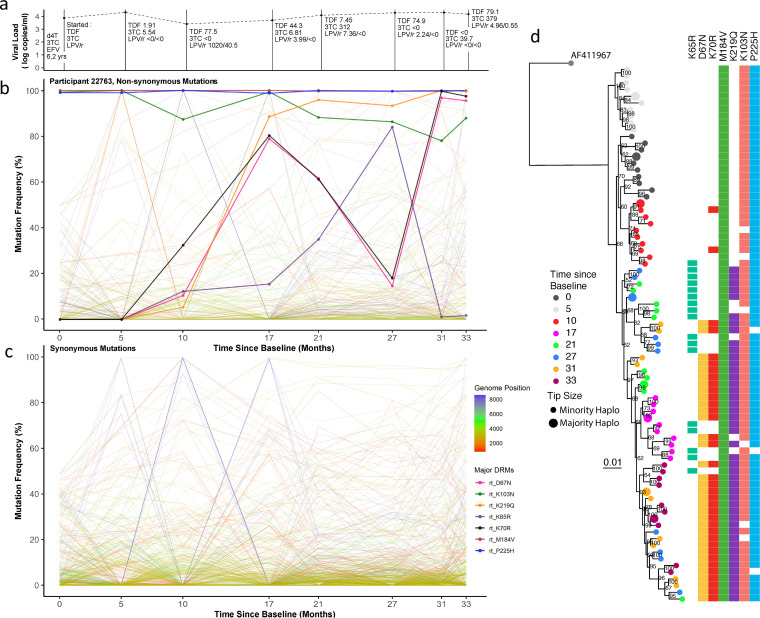
Drug regimen, adherence, and viral dynamics of patient 22763. (a) Viral load and regimen adherence. At successive time points the regimen was noted and plasma drug concentration was measured by HPLC (nmol/L). The patient had therapeutic levels of drug at several time points (3, 5, and 8), indicating variable adherence to the prescribed drug regimen. (b) Drug resistance- and non-drug resistance-associated nonsynonymous mutation frequencies. The patient had numerous drug resistance mutations in dynamic flux. Between time points 4 and 7, there was a complete population shift, indicated by reciprocal competition between the RT mutation K65R and the TAMs K67N and K70R. (c) Synonymous mutations frequencies. All mutations with a frequency of <10% or >90% at two or more time points were followed over successive time points. Several *env* mutations mimicked the nonsynonymous shifts observed between time points 2 and 4, suggestive of linkage. (d) Maximum likelihood phylogeny of reconstructed haplotypes. Time points 1 to 4 were found in distinct lineages. In later time points, from 5 to 8, haplotypes became more intermingled while maintaining antagonism between K65R- and K67N-bearing viruses.

**FIG 6:**
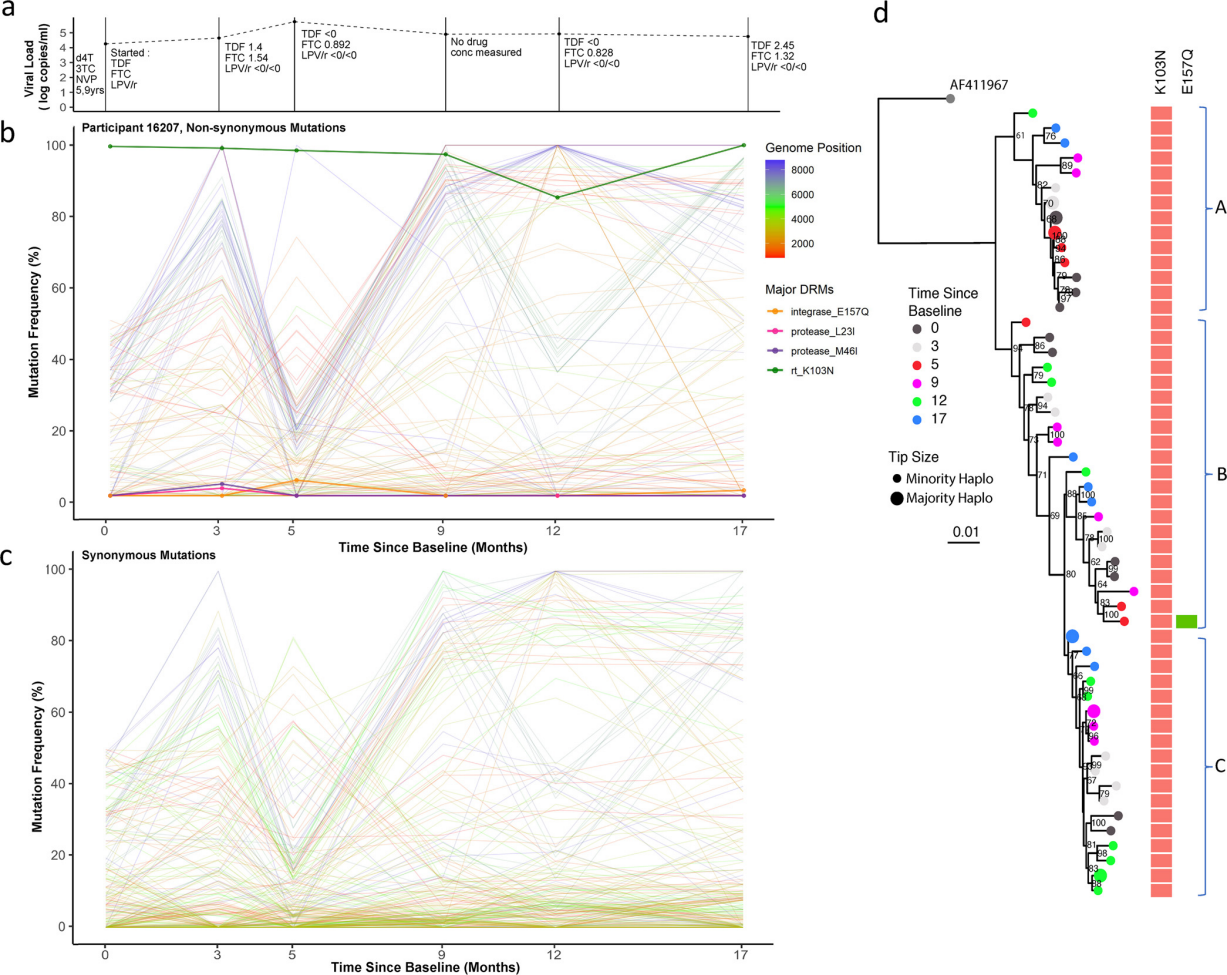
Drug regimen, adherence, and viral dynamics within patient 16207. (a) Viral load and drug levels. At successive time points the regimen was noted and the plasma drug concentration measured by HPLC (nmol/L). The patient displayed ongoing viremia and poor adherence to the prescribed drug regimen. (b) Drug resistance- and non-drug resistance-associated nonsynonymous mutation frequencies. The patient had only one major RT mutation, K103N, for the duration of the treatment period. Several antagonistic nonsynonymous switches predominantly in *env* were observed between time points 1 and 4. (c) Synonymous mutation frequencies. All mutations with a frequency of <10% or >90% at two or more time points were followed over successive time points. In contrast to nonsynonymous mutations, most synonymous changes were in *pol*, indicative of linkage to the *env* coding changes. (d) Maximum likelihood phylogeny of reconstructed haplotypes. Haplotypes were again clearly divided intro three distinct clades; each clade contained haplotypes from all time points, suggesting a lack of hard selective sweeps and intermingling of viral haplotypes with softer sweeps. Most viral competition occurred outside drug pressure.

In the absence of major PI mutations, we first examined nonsynonymous mutations across the whole genome (Fig. 4 and 5), with a specific focus on *pol* (to identify known first- and second-line NRTI-associated mutations) and *gag* (given its known involvement in PI susceptibility). We and others have previously shown that *gag* mutations accumulate during nonsuppressive PI therapy (34, 35). There are also data suggesting associations between *env* mutations and PI exposure (36, 37). Tables S1 to S3 summarize the changes in variant frequencies of *gag*, *pol*, and *env* mutations in patients over time. We found between two and four mutations at sites previously associated with PI resistance in each patient, all at persistently high frequencies (>90%) even in the absence of presumed drug pressure. This is explained by the fact that a significant proportion of sites associated with PI exposure are also polymorphic across HIV-1 subtypes (20, 38). To complement this analysis, we examined underlying synonymous mutations across the genome. This revealed complex changes in the frequencies of multiple nucleotide residues across all genes. These changes often formed distinct chevron-like pattens between time points (Fig. 4c and 6b), indicative of linked alleles dynamically shifting, which is in turn suggestive of competition between viral haplotypes.

Three patients (15664, 16207, and 22763), which had the greatest number of time points for ongoing comparison and had the highest read coverage, were selected for in-depth viral dynamics analysis as discussed below.

**Patient 15664.** This patient had consistently low plasma concentrations of all drugs at each measured time point, with detectable levels measured only at month 15 and beyond (Fig. 4a). At baseline, while on NNRTI-based 1st-line ART, known NRTI (M184V) and NNRTI (K103N and P225H) DRMs (5) were at high prevalence in the virus populations, which is as expected while adhering to 1st-line treatments. Haplotype reconstruction and subsequent analysis inferred the presence of a majority haplotype carrying all three of these mutations at baseline, as well as a minority haplotype with the absence of P225H (Fig. 4d, dark gray circles). Following the switch to a 2nd-line regimen, variant frequencies of M184V and P225H dropped below detection limits (<2% of reads), while K103N remained at high frequency (Fig. 4b). Haplotype analysis was concordant, revealing that viruses with K103N, M184V, and P225H were replaced by haplotypes with only K103N (Fig. 4d, light gray circles). At time point 2 (month 8), there were also numerous synonymous mutations observed at high frequency in both *gag* and *pol* genes, corresponding with the switch to a 2nd-line regimen. At time point 3 (15 months post-switch to 2nd-line regimen) drug concentrations were highest, though still low in absolute terms, indicating poor adherence. Between time points 3 and 4 we observed a 2-log reduction in viral load, with a modest change in frequency of RT DRMs. However, we observed synonymous variant frequency shifts predominantly in both *gag* and *pol* genes, as indicated by multiple variants increasing and decreasing contemporaneously, creating characteristic chevron patterning (Fig. 4b). However, many of the changes were between intermediate frequencies, (e.g., between 20% and 60%), which differed from changes between time points 1 and 2, where multiple variants changed more dramatically in frequency from <5% to more than 80%, indicating harder selective sweeps. These data are in keeping with a soft selective sweep between time points 3 and 5. Between time points 5 and 6, the final two samples, there was another population shift; M184V and P225H frequencies fell below the detection limit at time point 6, whereas the frequency of K103N dropped from almost 100% to around 80% (Fig. 4b). This was consistent with the haplotype reconstruction, which inferred a dominant viral haplotype at time point 6 bearing only K103N, as well as three minor haplotypes with no DRMs at all (Fig. 4d, light blue circles).

The phylogeny of inferred haplotype sequences showed that haplotypes from all time points were interspersed throughout the tree (except at time point 4, which remained phylogenetically distinct). DRMs showed some segregation by clade; viruses carrying a higher frequency of DRMs (M184V, P225H, and K238T) were observed in clade A (Fig. 4d), and those with either K103N alone or no DRMs were preferentially located in clade C (Fig. 4d). However, this relationship was not clear-cut and therefore was consistent with competition between haplotypes during low drug exposure. Soft sweeps were evident, given the increasing diversity (Fig. 1, Fig. S4) of this patient.

**Patient 16207.** Viral loads in this patient were consistently above 10,000 copies/mL (Fig. 6a). As with patient 15664, detectable drug concentrations in blood plasma were either extremely low or absent at each measured time point, consistent with nonadherence to the prescribed regimen. There was little change in the frequency of DRMs throughout the follow-up period, even when making the switch to the 2nd-line regimen. NNRTI resistance mutations such as K103N are known to have minimal fitness costs (26) and can therefore persist in the absence of NNRTI pressure. Throughout treatment, the viruses from this patient maintained K103N at a frequency of >85% but also carried an integrase strand transfer inhibitor (INSTI)-associated mutation (E157Q) and PI-exposure-associated amino acid replacements (L23I and M46I) at low frequencies at time points 2 and 3. Despite little change in DRM site frequencies, very significant viral population shifts were observed at the whole-genome level, again indicative of selective sweeps (Fig. 6b and c). Between time points 1 and 4, several linked mutations changed abundance contemporaneously, generating chevron-like patterns of nonsynonymous changes in *env* specifically (blue lines, Fig. 6b). A large number of alleles increased in frequency from <40% to >80% at time point 1, followed by decreases in frequency from >70% to <30% at time point 3. Whereas large shifts in *gag* and *pol* alleles also occurred, the mutations involved were almost exclusively synonymous (red and green lines).

Phylogenetic analysis of inferred whole-genome haplotypes again showed a distinct cladal structure as observed in patient 15664 (Fig. 6d), although the dominant haplotypes were equally observed in the upper clade (A) and lower clade (C) (Fig. 6d). K103N was the majority DRM at all time points, except for a minority haplotype at time point 3, also carrying E157Q. Haplotypes did not cluster by time point. Significant diversity in haplotypes from this patient was confirmed by MDS (Fig. S4).

**Patient 22763.** This patient was notable for a number of large shifts in variant frequencies across multiple drug resistance-associated residues and synonymous sites. The drug plasma concentration for different drugs was variable yet detectable at most measured time points. This suggests that the patient took some of their prescribed drugs throughout the follow-up period (Fig. 5a). Non-PI DRMs such as M184V, P225H, and K103N were present at baseline (time of switch from first- to second-line treatments). These mutations persisted despite synonymous changes between time points 1 and 2. Most of the highly variable synonymous changes in this patient were found in the *gag* and *pol* genes (as in patient 16207) (Fig. 5c), but in this case *env* displayed large fluctuations in synonymous and nonsynonymous allelic frequencies over time. At time point 3, therapeutic concentrations of boosted lopinavir (LPV/r) and tenofovir (TDF) were measured in plasma and haplotypes clustered separately from the first two time points (Fig. 5d, light and dark gray circles). NGS confirmed that the D67N, K219Q, K65R, L70R, and M184V DRMs and NNRTI resistance mutations were present at low frequencies from time point 3 onward. Of note, between time points 3 and 6, therapeutic concentrations of TDF were detectable and coincided with increased frequencies of the canonical TDF DRM, K65R (5). The viruses carrying K65R outcompeted those carrying the thymidine analogue mutants (TAMs) D67N and K70R, while the lamivudine (3TC)-associated resistance mutation, M184V, persisted throughout. In the final three time points M46I emerged in protease but never increased in frequency above 6%. At time point 7, populations shifted again, with some haplotypes resembling those previously seen in time point 4, with D67N and K70R again being predominant over K65R in reverse transcriptase (Fig. 5d, green and blue circles). At the final time point (8) the frequency of K103N was approximately 85%, and the TAM-bearing populations continued to dominate over the K65R population, which at this time point had a low frequency.

Although the DRM profile suggested the possibility of a selective sweep, we observed the same groups of other nonsynonymous or synonymous alleles exhibiting dramatic frequency shifts, but to a lesser degree than in patients 16207 and 15664; i.e., chevron patterns were less pronounced, outside the *env* gene (Fig. 5b and c). Variable drug pressures placed on the viral populations throughout the 2nd-line regimen appear to have played some role in limiting haplotype diversity. Time points 1 to 4 all formed distinct clades, without intermingling, indicating that competition between populations was not occurring to the same degree as in previous patients. Some inferred haplotypes had K65R and others, the TAMs D67N and K70R. K65R was not observed in combination with D67N or K70R, consistent with previously reported antagonism between K65R and TAMs, whereby these mutations are not commonly found together within a single genome (39–41). One explanation for the disconnect between the trajectories of DRM frequencies over time and haplotype phylogeny is competition between different viral populations. Alternatively, emergence of haplotypes from previously unsampled reservoirs with different DRM profiles is possible, but one might have expected other mutations to characterize such haplotypes that would manifest as changes in the frequencies of large numbers of other mutations.

## DISCUSSION

The proportion of people living with HIV (PLWH) who are accessing ART has increased from 24% in 2010, to 68% in 2020 (42, 43). However, with the scale-up of ART, there has also been an increase in both pretreatment drug resistance (PDR) (44, 45) and acquired drug resistance (14, 46) to 1st-line ART regimens containing NNRTIs. Integrase inhibitors (specifically dolutegravir) are now recommended for first-line regimens by the WHO in regions where PDR exceeds 10% (47). Boosted PI-containing regimens remain 2nd-line drugs following 1st-line failure, though one unanswered question relates to the nature of viral populations during failure on PI-based ART, where major mutations in protease, described largely for less potent PIs, have not emerged. Here, we have comprehensively analyzed viral populations present in longitudinally collected plasma samples of chronically infected HIV-1 patients under nonsuppressive 2nd-line ART.

With the vast majority of PLWH who have been treated in the post-ART era, virus dynamics during nonsuppressive ART are important to understand, as there may be implications for future therapeutic success. For example, broadly neutralizing antibodies (bNab) are being tested not only for prevention, but also as part of remission strategies in combination with latency reversal agents. We know that HIV sensitivity to bNabs is dependent on *env* diversity (48, 49), and therefore prolonged ART failure with viral diversification could compromise sensitivity to these agents.

Our understanding of virus dynamics largely stems from studies that were limited to untreated individuals (12), with mostly subgenomic data analyzed rather than whole genomes (12). Traditional analyses of quasispecies distributions, for example, as reported by Yu et al. (50), suggest that viral diversity increases in longitudinal samples. However, the findings of Yu et al. were based entirely on short-read NGS data without considering whole-genome haplotypes. The added benefit of examining whole genomes is that linked mutations can be identified statistically using an approach that we recently developed (31). Indeed, haplotype reconstruction has proved beneficial in the analysis of compartmentalization and diversification of several RNA and DNA viruses, including HIV-1, cytomegalovirus (CMV), and SARS-CoV-2 (34, 51, 52).

Key findings of this study were, first, that diversity as defined by the number of quasispecies in each sample typically increased over time. Considering divergence, (a measure at the consensus level of how many mutations have accumulated in a current sequence, from the founder infection) in contrast to previous literature which showed that there was a degree of reversion to the founder strain (9), we show that there was no significant reversion in our study population. There was also no significant divergence from baseline or ancestral C consensus sequences when considering the whole genome. However, when considering 1,000-bp fragments of the genome in a sliding window, several regions in *pol*, *vpu*, and *env* significantly diverged from the consensus C sequence. Using 1,000-bp windows, we were unable to identify any large-scale reversions. This is consistent with the divergence hypothesis, as if large-scale reversion was an accepted phenomenon, then HIV-1 would eventually converge to a homogeneous sequence, rather than what we have presented in the manuscript.

A second key finding in our study was that synonymous mutations were generally 2- to 3-fold more frequent than nonsynonymous mutations during nonsuppressive ART during chronic infection—a finding in contrast to that seen previously in a longitudinal study of untreated individuals (2, 12, 50). Nonsynonymous changes were enriched in known polymorphic regions such as *env*, whereas synonymous changes were more often observed to fluctuate in the conserved *pol* gene. This finding may reflect early versus chronic infection and differing selective pressures. Haplotype reconstruction revealed evidence for competing haplotypes, with phylogenetic evidence for numerous soft selective sweeps in that haplotypes intermingled during periods when there were low drug concentrations measured in the blood plasma samples of patients. Nonadherence to drug regimens therefore offers opportunity for the HIV-1 reservoir to increase in size and is associated with higher levels of residual viremia (53), preventing future viral suppression due to accumulation and maintenance of beneficial mutations.

Individuals in the present study were treated with ritonavir-boosted lopinavir along with two NRTIs (typically tenofovir plus emtricitabine). We observed significant changes in the frequencies of NRTI mutations in two of the three patients studied in-depth. We saw evidence for possible archived virus populations with DRMs emerging during follow-up, in that large changes in DRM frequency were not always accompanied by changes at other sites. This is consistent both with the occurrence of soft selective sweeps and with previous observations that non-DRMs do not necessarily drift with other mutations to fixation (23). As frequencies of RT DRMs did not always segregate with haplotype frequencies (i.e., the same mutations were repeatedly observed on different genetic backgrounds), we suggest that a high number of recombination events, known to be common in HIV infections, were likely contributing to the observed haplotypic diversity.

Although no patient developed major resistance mutations to PIs at consistently high frequencies (https://hivdb.stanford.edu/dr-summary/resistance-notes/PI/), we did observe nonsynonymous mutations in *gag* which have been previously associated with mediating resistance to PI. There was, however, no temporal evidence of specific mutations being associated with selective sweeps. For example, PI exposure-associated residues in matrix (positions 76 and 81) were observed in patient 16207 prior to PI initiation (54). Furthermore, patient 16207 was one of two patients who achieved low-level viremic suppression (45 to 999 copies/mL) of viral replication at one or two time points. After both of these partial suppressions, the rebound populations appeared to be less diverse, consistent with drug-resistant viruses reemerging.

Mutations at sites in the HIV genome that are further apart than 100 bp are subject to frequent shuffling via recombination (55). Unlike the smooth LD decay curves for pairs of HIV mutations reported in the literature, we identified complex LD decay patterns within the genomes of viruses from individual patients—patterns indicative of nonrandom recombination. Recombination appears as the loss and gain of common genomic regions over successive time points between each patient’s haplotype populations (Fig. 3b). Viruses from patient 15664 with interhaplotype recombination events detectable in the *vif* and *vpr* genes were present at four of the six analyzed time points. In contrast, viruses in patient 22763 that had evidence of interhaplotype recombination events in the *gag*-*pol* genes were present at three of the eight analyzed time points. We explain these recombination events detectable in longitudinally sampled sequences, as reflected in the previously discussed chevron patterns whereby variants increase and subsequently decrease between time points. HIV quasispecies foster a degree of genetic diversity that facilitates rapid adaptive evolution through recombination whenever there exists within the quasispecies combinations of mutations that provide fitness advantages (8). The relationship between recombination and the accumulation of multiple DRMs within individual genomes is not clearly evident within the analyzed sequence data sets, with viruses sampled from each patient showing unique patterns of recombination. Interhaplotype recombinants detected at time points 2 and 6 in patient 16207 had recombination events in *pol* that involved the transfer of the major DRM, K103N. Three independent inter-haplotype recombination events detected in the *pol* gene from patient 22763 at time points 2, 4, and 6 resulted in no change in DRMS at time point 2, gain of DRMS at time point 4, or loss of DRMs at time point 6. The recombination dynamics in this patient were occurring against a backdrop of apparent antagonism between TAMs and DRMs (K65R and D67N). Finally, patient 15664 steadily lost DRMs throughout the longitudinal sampling period, although we found no evidence of recombination being implicated in this loss. This suggests that, in the absence of strong drug pressures, viral populations only maintained DRMs which were crucial for providing resistance to drugs that the patient was variably adhering to at the time.

Phylogenetic analyses of whole-genome viral haplotypes demonstrated two common features: (i) evidence for selective sweeps following therapy switches or large changes in plasma drug concentrations, with hitchhiking of synonymous and nonsynonymous mutations, and (ii) competition between multiple viral haplotypes that intermingled phylogenetically alongside soft selective sweeps. The diversity of viral populations was maintained between successive time points with ongoing viremia, particularly in *env*. Changes in haplotype dominance were often distinct from the dynamics of drug resistance mutations in reverse transcriptase (RT), indicating the presence of softer selective sweeps and/or recombination.

This study had some limitations; we examined eight patients with ongoing viremia and variable adherence to 2nd-line drug regimens, with three of these being examined in-depth. Despite the small sample size, this type of longitudinal sampling of ART-experienced patients is unprecedented. We are confident that the combination of computational analyses has provided a detailed understanding of viral dynamics under nonsuppressive ART that will be applicable to wider data sets. The method used to reconstruct viral haplotypes *in silico* is novel and has previously been validated in HIV-1-positive patients coinfected with CMV (51). We are confident that the approach implemented by HaROLD has accurately, if conservatively, estimated haplotype frequencies, and future studies should look to validate these frequencies using an *in vitro* method such as single-genome amplification.

Despite there being high viral loads present at each of the analyzed time points, nuances of the sequencing method resulted in suboptimal gene coverage, particularly in the *env* gene. To ensure that uneven sequencing coverage did not bias our analyses, we ensured that variant analysis was only performed where coverage was >100 reads. We also utilized a second method of haplotype reconstruction, in order to determine concordance of DRM calls between the two methods used. We find that there was good concordance between the two methods, specifically highlighted by the antagonism between TAMs (D67N and K70R) and NRTI mutations (K65R) in patient 22763 (Fig. S6).

10.1128/mbio.00269-22.6 FIG S6 Maximum likelihood phylogenies of reconstructed haplotypes using Cliquesnv. As this method of haplotype reconstruction does not consider the longitudinal aspect of the data, haplotypes are segregated according to time point. DRMs are largely consistent with those inferred by HaROLD. Download FIG S6, PDF file, 0.2 MB. © Crown copyright 2022. 2022 Crown https://creativecommons.org/licenses/by/4.0/ This content is distributed under the terms of the Creative Commons Attribution 4.0 International license.

In summary, we have found compelling evidence of HIV-1 within-host viral diversification, recombination, and haplotype competition during nonsuppressive ART. In the future, patients failing PI-based regimens are likely to be switched to INSTI-based ART (specifically dolutegravir in South Africa) prior to genotypic typing or resistance analysis. Although the prevalence of underlying major INSTI resistance mutations is low in sub-Saharan Africa (56, 57), data linking individuals with NNRTI resistance with poorer virological outcomes on dolutegravir (58), coupled with a history of intermittent adherence, warrant further investigation. Having shown that long-time intrahost PI failure increases the intrapatient diversity of HIV populations, monitoring future drug-failure cases will be of interest due to their capacity to maintain a reservoir of transmissible drug-resistant viruses, as well as impacting responses to future therapies.

## MATERIALS AND METHODS

### Study and patient selection.

This cohort was nested within the French ANRS 12249 Treatment as Prevention (TasP) trial (27). TasP was a cluster-randomized trial comparing an intervention arm which offered ART after HIV diagnosis irrespective of patient CD4^+^ count to a control arm offering ART according to prevailing South African guidelines. In total, a subset of 44 longitudinal samples from eight chronically infected patients with virological failure of 2nd-line PI-based ART, with viremia above 1,000 copies/mL, were analyzed. From these eight patients, three patients with a mean coverage of >2,000 reads across the whole genome were selected from in-depth viral dynamic analysis. All samples were collected from blood plasma. The Illumina MiSeq platform and an adapted protocol for sequencing were used (59). Adherence to 2nd-line regimens was measured by high-performance liquid chromatography (HPLC) using plasma concentration of drug levels as a proxy. Drug levels were measured at each time point with detectable viral loads, post-PI initiation. Cutoffs for assessment of adherence were selected from published literature.

Ethical approval was originally granted by the Biomedical Research Ethics Committee (BFC 104/11) at the University of KwaZulu-Natal and the Medicines Control Council of South Africa for the TasP trial (Clinicaltrials.gov: NCT01509508; South African Trial Register: DOH-27-0512-3974). The study was also authorized by the KwaZulu-Natal Department of Health in South Africa. Written informed consent was obtained from all patients. Original ethical approval also included downstream sequencing of blood plasma samples and analysis of those sequences to better understand drug resistance. No additional ethical approval was required for this.

### Illumina sequencing.

Sequencing of viral RNA was performed as previously described by Derache et al. (60) using a modified protocol previously described by Gall et al. (61). Briefly, RNA was extracted from 1 mL of plasma with a detectable viral load of >1,000 copies/mL, using QIAamp viral RNA minikits (Qiagen, Hilden, Germany) and eluted in 60 μL of elution buffer. The near-full HIV genome was amplified with four HIV-1 subtype C primer pairs, generating 4 overlapping amplicons of between 2,100 and 3,900 kb.

DNA concentrations of amplicons were quantified with the Qubit double-stranded DNA (dsDNA) high-sensitivity (HS) assay kit (Invitrogen, Carlsbad, CA). Diluted amplicons were pooled equimolarly and prepared for the library using the Nextera XT DNA library preparation and the Nextera XT DNA sample preparation index kits (Illumina, San Diego, CA), following the manufacturer’s protocol.

### Genomics and bioinformatics.

Poor-quality reads (with a Phred score of <30) and adapter sequences were trimmed from FastQ files with TrimGalore! v0.6.519 (62) and mapped to a dual-tropic, clade C, South African reference genome (GenBank accession number AF411967) with Minimap2 (63). The reference genome was manually annotated in Geneious Prime v2020.3 with DRMs according to the Stanford HIV Drug Resistance Database (HIVdb) (64). Optical PCR duplicate reads were removed using Picard tools (http://broadinstitute.github.io/picard). Finally, Qualimap 2 (65) was used to assess the mean mapping quality scores and coverage in relation to the reference genome for the purpose of excluding poorly mapped sequences from further analysis. Single nucleotide polymorphisms (SNPs) were called using VarScan 2 (66) with a minimum average quality of 20, minimum variant frequency of 2%, and in at least 100 reads. These were then annotated by gene, codon, and amino acid alterations using an in-house script (67) modified to utilize HIV genomes.

All synonymous and nonsynonymous variants (including DRMs) were examined, and their frequencies were compared across successive time points. Synonymous variants were excluded from analysis if their prevalence remained at ≤10% or ≥90% across all time points. DRMs were retained for analysis if they were present at over 2% frequency and on at least two reads. A threshold of 2% is supported by a study evaluating different analysis pipelines, which reported fewer discordances over this cutoff (68).

### Measuring divergence or reversion to baseline and consensus C ancestor.

For each patient, divergence over time from inferred founder state was measured for (i) the baseline sequence for each patient and (ii) a reconstructed subtype C consensus. The full-length HIV-1 subtype C consensus was downloaded from the LANL HIV database, and annotations from the subtype C reference sequence (GenBank accession number AF411967) used for haplotype reconstruction were transferred to this genome using Geneious Prime v2021.1.0 to ensure that positions remained consistent throughout.

Divergence was measured as the pairwise distance between time point consensus and founder, calculated using the dist.dna() package with a TN93 nucleotide-nucleotide substitution matrix and with pairwise deletion as implemented in the R package Ape v5.4.

As a validation, an in-house script was used to examine all amino acids on a site-per-site basis. The initial AA at time point 1 (baseline or ancestral C) is recorded. Where there is a mutation at any subsequent time point except the last time point, we measure if the AA at the final time point is the same or different from the first. If there has been a reversion mutation, this will be the same as the first time point. If there has been a diverging mutation, this will be different from the first.

### Linear mixed-effects models.

To investigate the general relationship of time in months to divergence, incorporating all 8 patients, we built a series of linear mixed-effects models implemented in the lmer R package. Divergence was treated as the response, time was treated as a fixed effect, and patient was treated as a random effect. We built similar models for the whole-genome and discrete genomic portions analysis for each founder strain. We tested if time had a non-0 effect on divergence by calculating the *P* value using Satterthwaite’s method as implemented in the lmerTEst package. For the 1,000-bp analyses, a Benjamini-Hochberg correction adjustment was undertaken to account for 9 tests within the same sample.

### Haplotype reconstruction and phylogenetics.

Whole-genome viral haplotypes were constructed for each patient time point using HaROLD (Haplotype Reconstruction for Longitudinal Samples) (31). The first stage consists of SNPs being assigned to each haplotype such that the frequency of variants is equal to the sum of the frequencies of haplotypes containing a specific variant. This considers the frequency of haplotypes in each sample, the base found at each position in each haplotype, and the probability of erroneous measurements at that site. Maximal log likelihood was used to optimize time-dependent frequencies for longitudinal haplotypes, which was calculated by summing over all possible assignments of haplotype variants. Haplotypes were then constructed based on posterior probabilities.

After constructing haplotypes, a 2nd stage or refinement process remaps reads from BAM files to constructed haplotypes. This begins with the *a posteriori* probability of each base occurring at each site in each haplotype from the first stage but relaxes the assumption that haplotypes are identical at each sample time point and instead uses variant colocalization to refine haplotype predictions. Starting with the estimated frequency of each haplotype in a sample, haplotypes are optimized by probabilistically assigning reads to the various haplotypes. Reads are then reassigned iteratively until haplotype frequencies converge. The number of haplotypes either increases or decreases as a result of combination or division according to Akaike information criterion (AIC) scores, in order to present the most accurate representation of viral populations at each time point.

Whole-genome nucleotide diversity was calculated from BAM files using an in-house script (https://github.com/ucl-pathgenomics/NucleotideDiversity). Briefly diversity is calculated by fitting all observed variant frequencies to either a beta distribution or four-dimensional Dirichlet distribution plus delta function (representing invariant sites). These parameters were optimized by maximum log likelihood.

Maximum-likelihood phylogenetic trees and ancestral reconstruction were performed using IQ-TREE 2 v2.1.3 (69) and a GTR+F+I model with 1,000 ultrafast bootstrap replicates (70). All trees were visualized with Figtree v1.4.4 (https://github.com/rambaut/figtree/releases), rooted on the AF411967 reference sequence, and nodes were arranged in descending order. Phylogenies were manipulated and annotated using ggtrree v2.2.4.

Additionally, as a sensitivity analysis, Cliquesnv (71) was used to infer a second set of haplotypes using the following flags: -m snv-illumina -fdf extended4 -threads 20 -cm accurate. This was to determine concordance of drug resistance mutation calls within haplotypes.

### Multidimensional scaling (MDS) plots.

Pairwise distances between these consensus sequences were calculated using the dist.dna() package, with a TN93 nucleotide-nucleotide substitution matrix and with pairwise deletion implemented in the R package Ape v5.4. Nonmetric multidimensional scaling (MDS) was implemented using the metaMDS() function in the R package vegan v2.5.7. MDS is a method to attempt to simplify high-dimensional data into a simpler representation of reducing dimensionality while retaining most of the variation relationships between points. We find that like network trees, nonmetric MDS better represents the true relative distances between sequences, whereas eigenvector methods are less reliable in this sense. In a genomics context we can apply dimensionality reduction on pairwise distance matrices, where each dimension is a sequence with data points of *n* – 1 sequences pairwise distance. The process was repeated with whole-genome haplotype sequences.

### Linkage disequilibrium and recombination.

Starting with a sequence alignment, we determined the pairwise LD r^2^ associations for all variable sites using WeightedLD (72) without weighting. This method allowed us to easily exclude sites with any insertions or ambiguous characters, where we used the options –min-acgt 0.99 and –min-variability 0.05. The pairwise r^2^ values (the square of the correlation coefficient between two indicator variables) were then binned per 200-bp comparison distance blocks along the genome, and the mean r^2^ values were taken and represented graphically to assess LD decay. This analysis was run for the three patients taken forward for in-depth analysis and run using an alignment of all their time point samples. Graphics were generated using R v4.04.

We first performed an analysis for detecting individual recombination events in individual genome sequences using the RDP, GENECONV, BOOTSCAN, MAXCHI, CHIMAERA, SISCAN, and 3SEQ methods implemented in RDP5 (73) with default settings. Putative breakpoint sites were identified and manually checked and adjusted if necessary using the BURT method with the MAXCHI matrix and LARD two-breakpoint scan methods. Final recombination breakpoint sites were confirmed if at least three or more methods supported the existence of the recombination breakpoint.

### Data and code availability.

All BAM files used to undertake analyses have been deposited in the SRA database with the accession numbers SRR15510046 to SRR15510072. Custom code used to produce figures and graphs can be found at https://github.com/ojcharles/HIV1-evolutionary-dynamics.
