# Supplementary figures and images for: HIV-1 Evolutionary Dynamics under Nonsuppressive Antiretroviral Therapy
Source: mBio. 2022 Apr 21;13(3):e00269-22. doi: 10.1128/mbio.00269-22 (PMC9239331; doi:10.1128/mbio.00269-22)

Patient 15664

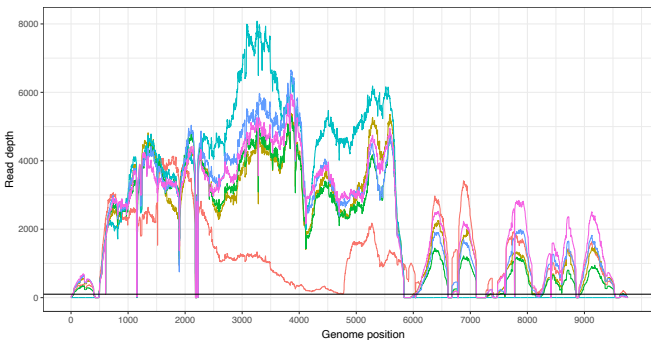

Patient 16207

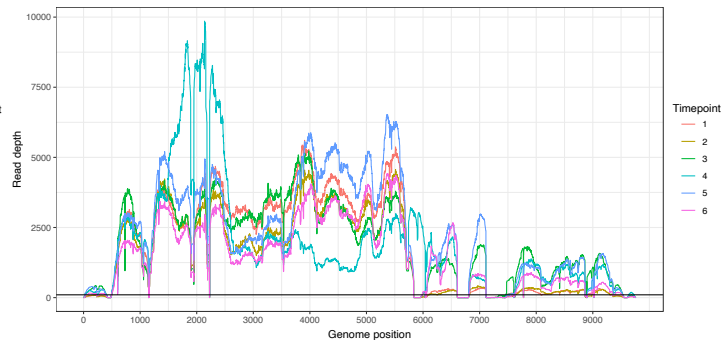

Patient 22763

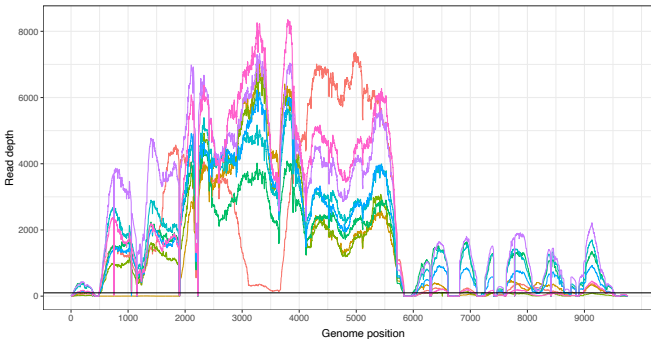

Patient 22828

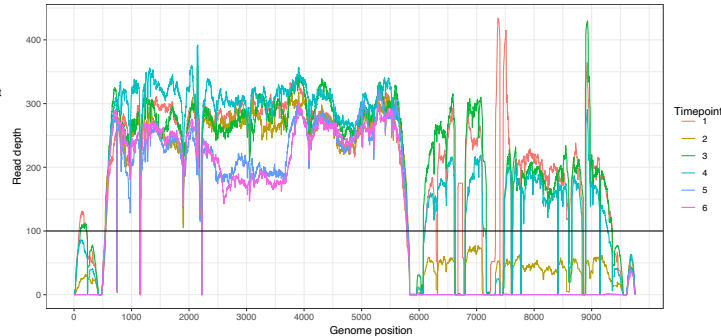

Patient 26892

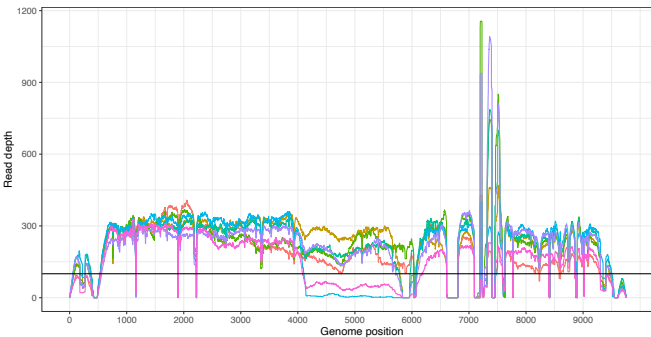

Patient 28545

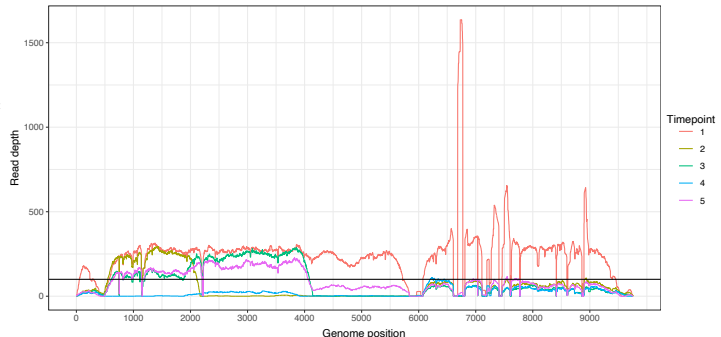

Patient 29447

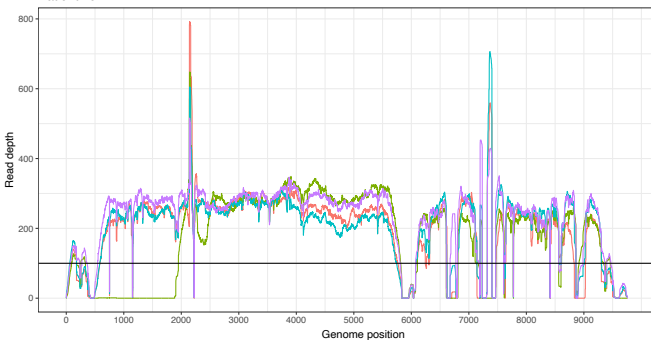

Patient 47939

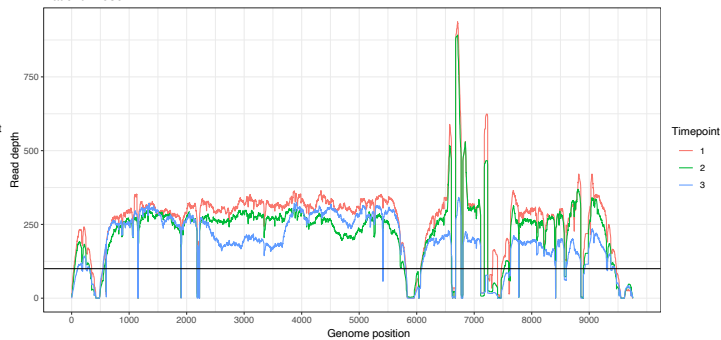

Supplement: FIG S1 [file mbio.00269-22-sf001.pdf]

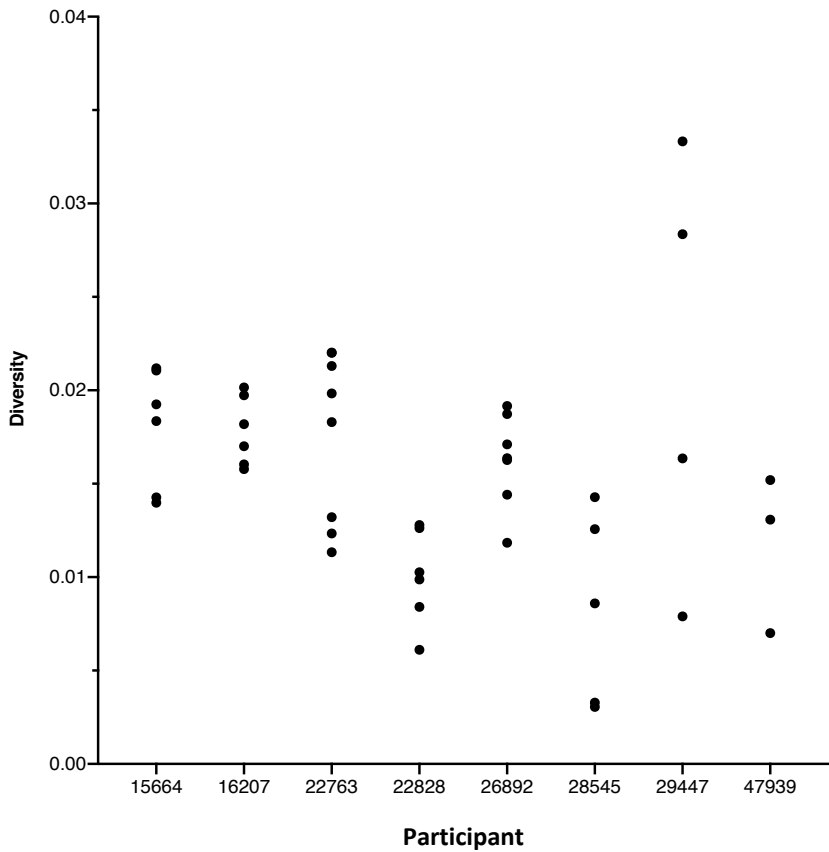

Supplement: FIG S2 [file mbio.00269-22-sf002.pdf]

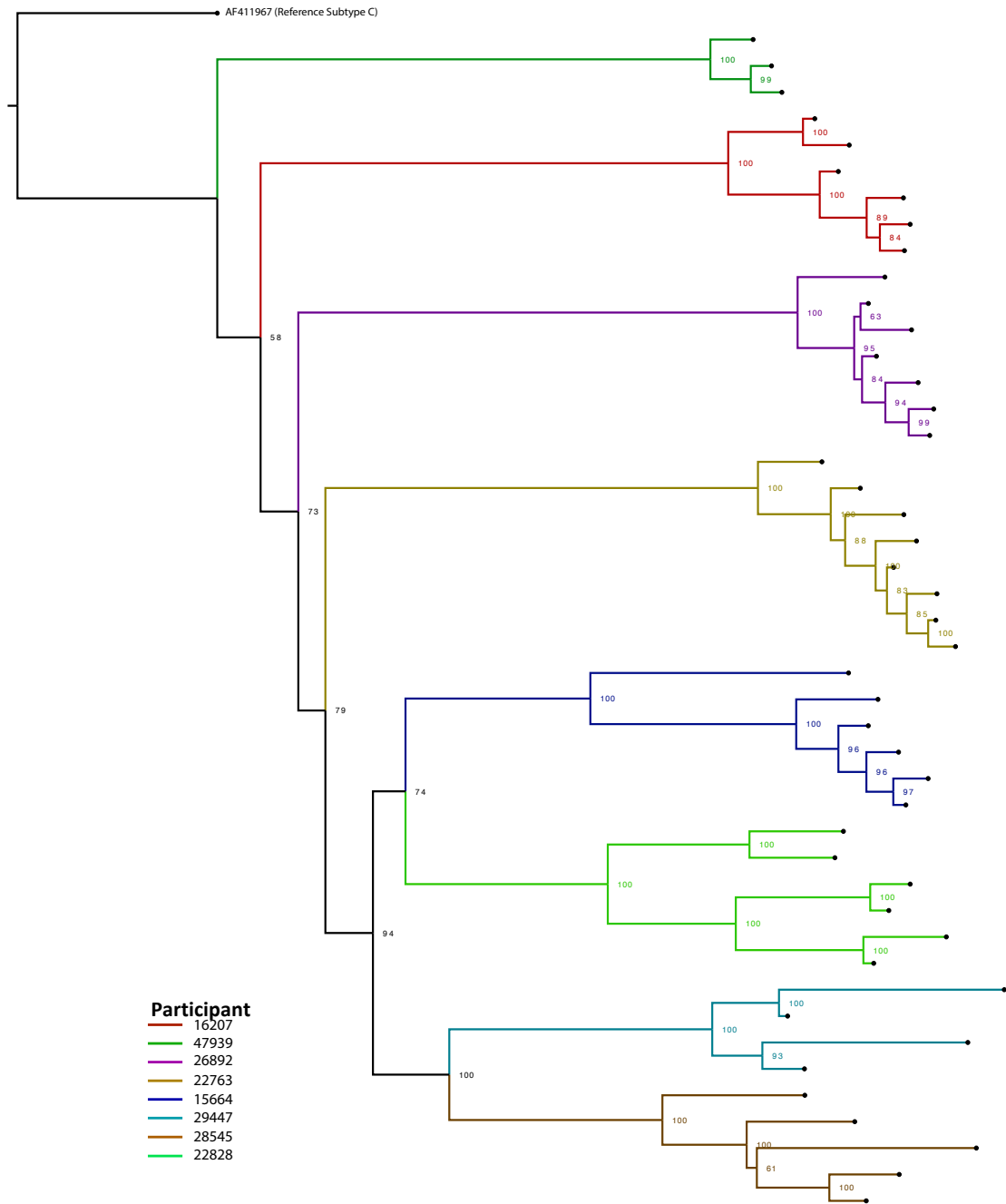

Supplement: FIG S3 [file mbio.00269-22-sf003.pdf]

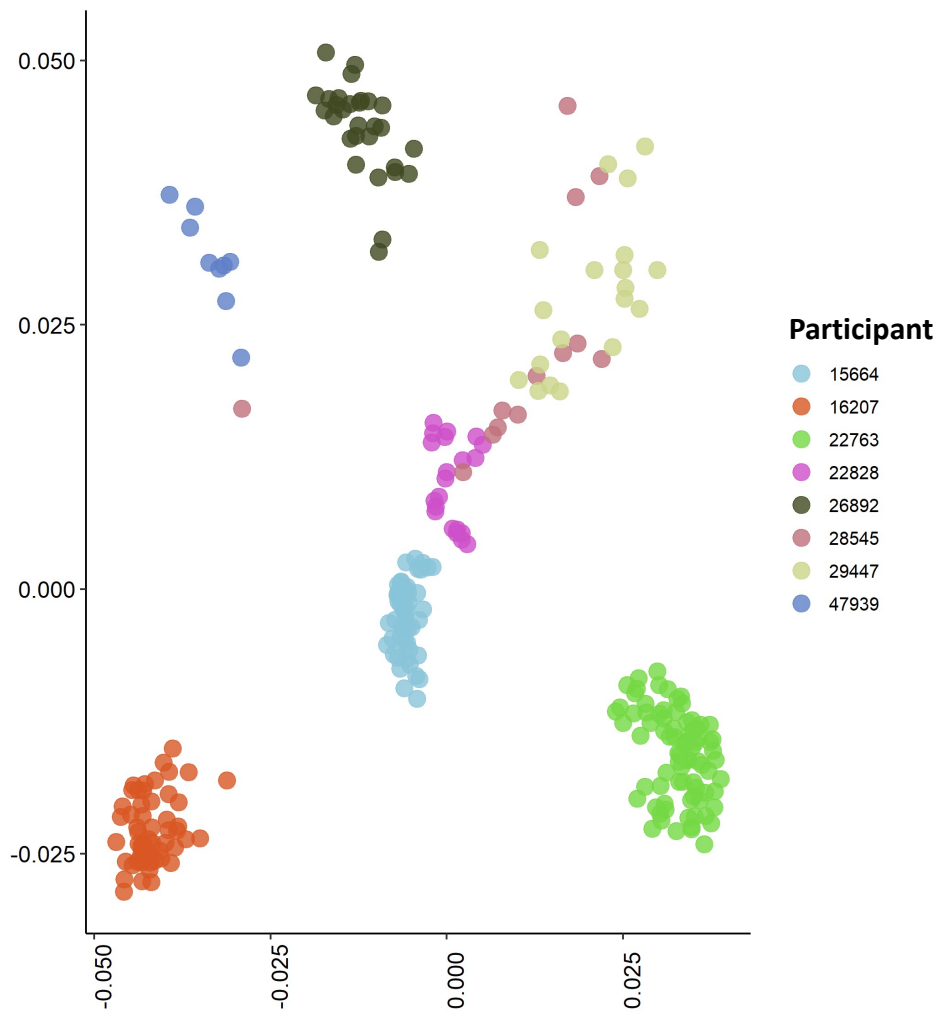

Supplement: FIG S4 [file mbio.00269-22-sf004.pdf]

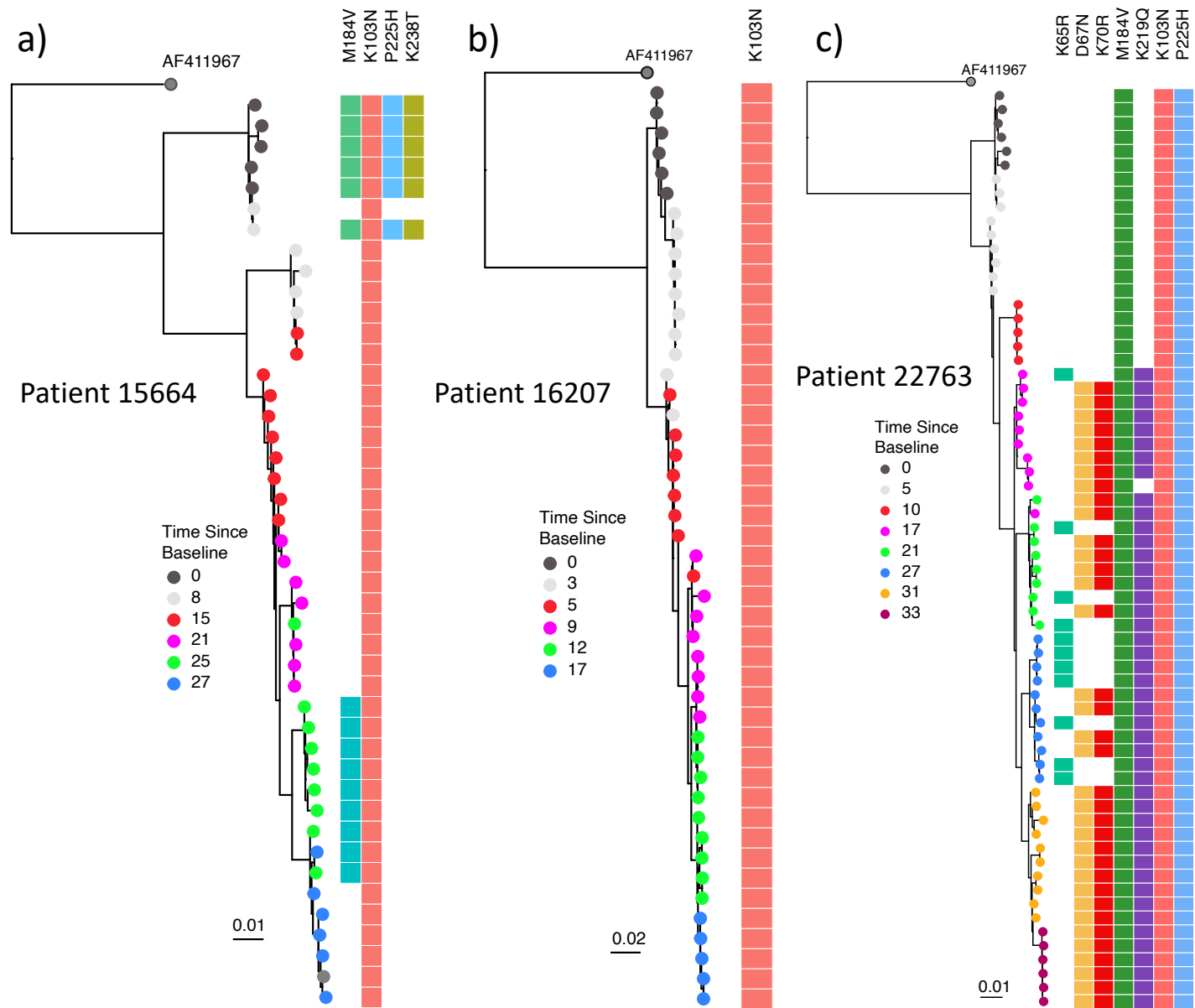

Supplement: FIG S6 [file mbio.00269-22-sf006.pdf]
