# Supplementary material for: HIV-1 Evolutionary Dynamics under Nonsuppressive Antiretroviral Therapy
Source: mBio. 2022 Apr 21;13(3):e00269-22. doi: 10.1128/mbio.00269-22 (PMC9239331; doi:10.1128/mbio.00269-22)
Supplement: TABLE S4 [file mbio.00269-22-st004.docx]

|  |  |  |
| --- | --- | --- |
| **Genomic position** | **P value (Benjamini-Hochberg FDR adjusted)** | |
|  | Founder <-> Baseline Sequence | Founder <-> Ancestral C |
|  |  |  |
| 1-1000 | 0.72 | 0.26 |
| 1001-2000 | 0.85 | 0.24 |
| 2001-3000 | 0.72 | 0.11 |
| 3001-3000 | 0.72 | 0.24 |
| 4001-5000 | 0.72 | 0.24 |
| 5001-6000 | 0.85 | 0.15 |
| 6001-7000 | 0.72 | 0.06 |
| 7001-8000 | 0.72 | 0.06 |
| 8001-9000 | 0.72 | 0.06 |

Significance tests of explanatory variable (time in months) viral divergence from two founder strains.

**Supplementary Table 4.** Results from linear mixed effects models of effect of months on divergence from founder virus. No values were significant (<0.05).
