# Supplementary material for: HIV-1 Evolutionary Dynamics under Nonsuppressive Antiretroviral Therapy
Source: mBio. 2022 Apr 21;13(3):e00269-22. doi: 10.1128/mbio.00269-22 (PMC9239331; doi:10.1128/mbio.00269-22)
Supplement: FIG S5 [file mbio.00269-22-sf005.pdf]

a)

Participant 15664 Genome Position

	3529	3533	3573	3550	3562	3578	3589	3619	4231	4243	4246	4263	6091	6100	6106	6112	6117
15664, Timepoint 1	G	C	A	C	G	C	G	G	T	A	T	A	G	C	A	T	A
15664, Timepoint 2	A	G	G	T	A	T	A	T	A	T	G	G	G	C	A	T	A
15664, Timepoint 3	A	G	G	T	A	T	A	T	T	A	T	A	G	C	A	T	A
15664, Timepoint 4	G	C	A	T	G	C	G	G	T	A	T	A	G	C	A	T	A
15664, Timepoint 5	A	G	G	T	A	T	A	T	A	T	G	G	G	C	A	T	A
15664, Timepoint 6	A	G	G	T	A	T	A	T	T	A	T	A	A	T	G	C	C

b)

Participant 16207 Genome Position

	2936	2980	3017	3092	3154	3325	3577	3589	3604	6847	6868	6923	6926	6928	6955
16207, Timepoint 1	G	T	T	T	T	G	T	A	G	C	C	A	G	G	G
16207, Timepoint 2	G	G	T	T	T	G	T	A	G	T	A	G	A	T	T
16207, Timepoint 3	G	T	T	T	T	G	T	A	G	C	C	A	G	G	G
16207, Timepoint 4	A	G	A	C	C	A	C	G	A	T	A	G	A	T	T
16207, Timepoint 5	A	G	A	C	C	A	C	G	A	C	C	A	G	G	G
16207, Timepoint 6	A	T	T	C	T	A	T	A	G	T	A	G	A	T	T

c)

Participant 22763 Genome Position

	2704	2711	2727	2732	2737	2742	2753	5849	5859	5861	5864	5879	5884	5885	5899
22763, Timepoint 1	T	G	A	G	T	A	T	C	T	T	A	T	A	T	C
22763, Timepoint 2	T	G	A	G	T	A	T	C	T	T	A	T	A	T	C
22763, Timepoint 3	T	G	A	G	T	A	T	C	T	T	A	T	A	T	C
22763, Timepoint 4	T	A	A	A	T	G	C	A	A	T	G	C	A	C	T
22763, Timepoint 5	T	A	A	A	T	G	C	A	A	T	G	C	A	C	T
22763, Timepoint 6	C	A	G	A	G	G	C	T	A	C	G	C	C	G	A
22763, Timepoint 7	T	A	A	A	T	G	C	A	A	T	G	C	A	C	T
22763, Timepoint 8	T	A	A	A	T	G	C	C	T	T	A	T	A	T	C
